# Effect of Prognostic Guided Management of Patients With Acute Pulmonary Embolism According to the European Society of Cardiology Risk Stratification Model

**DOI:** 10.3389/fcvm.2022.872115

**Published:** 2022-04-12

**Authors:** David Jiménez, Carmen Rodríguez, Beatriz Pintado, Andrea Pérez, Luis Jara-Palomares, Raquel López-Reyes, Pedro Ruiz-Artacho, Alberto García-Ortega, Behnood Bikdeli, José Luis Lobo, the IPEP investigators

**Affiliations:** ^1^Respiratory Department, Hospital Ramón y Cajal, Instituto Ramón y Cajal de Investigación Sanitaria IRYCIS, Madrid, Spain; ^2^Department of Medicine, Universidad de Alcalá, Madrid, Spain; ^3^CIBER Enfermedades Respiratorias, Instituto de Salud Carlos III, Madrid, Spain; ^4^Respiratory Department, Virgen del Rocío Hospital, Instituto de Biomedicina, Seville, Spain; ^5^Respiratory Department, Hospital La Fe, Valencia, Spain; ^6^Department of Internal Medicine, Clínica Universidad de Navarra, Madrid, Spain; ^7^Interdisciplinar Teragnosis and Radiosomics Research Group (INTRA-Madrid), Universidad de Navarra, Madrid, Spain; ^8^Cardiovascular Medicine Division, Brigham and Women’s Hospital, Harvard Medical School, Boston, MA, United States; ^9^Center for Outcomes Research and Evaluation, Yale School of Medicine, New Haven, CT, United States; ^10^Cardiovascular Research Foundation, New York, NY, United States; ^11^Respiratory Department, Hospital Araba, Vitoria-Gasteiz, Spain

**Keywords:** prognosis, LOS, mortality, pulmonary embolism, complications

## Abstract

**Background:**

A recent trial showed that management driven by prognostic assessment was effective in reducing the length of stay (LOS) for acute stable pulmonary embolism (PE). The efficacy and safety of this strategy in each subgroup of risk stratification remains unknown.

**Methods:**

We conducted a *post-hoc* analysis of the randomized IPEP study to evaluate the effect of a management strategy guided by early use of a prognostic pathway in the low- and intermediate-high risk subgroups defined by the European Society of Cardiology (ESC) model. These subgroups were retrospectively identified in the control arm. The primary outcome was LOS. The secondary outcomes were 30-day clinical outcomes.

**Results:**

Of 249 patients assigned to the intervention group, 60 (24%) were classified as low-, and 30 (12%) as intermediate-high risk. Among 249 patients assigned to the control group, 66 (27%) were low-, and 13 (5%) intermediate-high risk. In the low-risk group, the mean LOS was 2.1 (±0.9) days in the intervention group and 5.3 (±2.9) days in the control group (*P* < 0.001). In this group, no significant differences were observed in 30-day readmissions (0% vs. 3.0%, respectively), all-cause (0% vs. 0%) and PE-related mortality rates (0% vs. 0%), or severe adverse events (0% vs. 1.5%). In the intermediate-high risk group, the mean LOS was 5.3 (±1.8) days in the intervention group and 6.5 (±2.5) days in the control group (*P* = 0.08). In this group, no significant differences were observed in 30-day readmissions (3.3% vs. 3.0%, respectively), all-cause (6.7% vs. 7.7%) and PE-related mortality rates (6.7% vs. 7.7%), or severe adverse events (16.7% vs. 15.4%).

**Conclusion:**

The use of a prognostic assessment and management pathway was effective in reducing the LOS for acute PE without comprising safety across subgroups of risk stratification.

**Clinical Trial Registration:**

[ClinicalTrials.gov], Identifier [NCT02733198].

## Introduction

Pulmonary embolism (**PE**) is a common condition and a leading health issue worldwide with associated substantial morbidity and mortality (1, 2). PE affects about 400,000 people each year in Europe (3). In the United States, between 300,000 and 600,000 cases occur each year (4), which contribute to at least 40,000 deaths (5).

Pulmonary embolism is associated with considerable health care resource utilization (6). Much of the economic burden of venous thromboembolism (**VTE**) is related to hospitalization costs (7–10) and increasing the number of patients with PE treated as outpatients could potentially reduce PE-related hospital burdens. Although approximately 30% of patients with PE might be suitable for outpatient therapy of their disease (11), the vast majority is still admitted to the hospital (12, 13). In addition, despite recent trends indicating a decline in length of hospital stay (**LOS**) after PE diagnosis (14), duration of hospitalization is still high (15, 2). Therefore, validation of strategies aimed at safely reducing the LOS are of paramount importance.

For hospitalized patients with acute symptomatic PE, the recent Prognostication in acute Pulmonary Embolism (**IPEP**) trial showed that prognostication and use of objective criteria for mobilization and early hospital discharge to be safe and associated with reduction in downstream laboratory or echocardiographic testing, and that it was effective in reducing LOS by 2 days, compared with usual care (16). Since the trial used a risk stratification scheme and specific criteria for mobilization and hospital discharge in each subgroup of risk, it would be key to assess whether the efficacy and safety of the intervention was maintained among those patients who benefit most from early discharge (low-risk), and among those at highest risk for short-term complications (intermediate-high risk).

Using the results of a previous randomized trial, the objective of the current manuscript was to specifically assess the effect of a management strategy guided by early use of a prognostic pathway in the low- and intermediate-high risk subgroups defined by the European Society of Cardiology (**ESC**) model (17).

## Materials and Methods

We performed *post-hoc* analyses of the recently completed IPEP trial. IPEP was a multicenter, open-label, randomized, clinical trial aimed at evaluating whether a management strategy guided by early use of a prognostic pathway would be more effective than usual care in reducing LOS in hospitalized patients with acute PE. The rationale, design, and primary results of the IPEP study were described previously (16). The trial was conducted in nine academic hospitals across Spain. The institutional review board at each of the participating sites approved the protocol, and each patient provided written informed consent.

Briefly, patients hospitalized with hemodynamically stable acute PE were randomized to either a prognostic assessment and management pathway including risk stratification, followed by predefined criteria for mobilization and hospital discharge (intervention group) vs. usual care (control group). Per protocol, intervention for patients in the active arm was provided by trial investigators who were strictly advised to follow the protocol-recommended pathway, while management of patients in the control arm was performed by other clinicians according to their routine practice.

For patients in the intervention arm, trial investigators measured vital signs to calculate the simplified Pulmonary Embolism Severity Index (**sPESI**) (18). An sPESI score of 0 identified low-risk patients. Patients with a sPESI > 1 constituted an intermediate-risk group. Within this group, patients had to undergo troponin testing and, for those with a positive result, echocardiographic assessment for right ventricular (**RV**) dysfunction. Patients with an sPESI > 1 and abnormality for only troponin levels or only echocardiographic RV dysfunction (or neither) comprised the intermediate-low risk group. In turn, patients with an sPESI > 1 and both elevated troponin levels and echocardiographic RV dysfunction comprised the intermediate-high risk group (Supplementary Figure 1).

For the present analysis, only low- and intermediate-high risk patients were included, and prognostication of patients randomly assigned to usual care was done retrospectively.

### Outcomes

The primary outcome of the trial was the LOS, defined as the interval from diagnosis of PE at the emergency department to discharge from the hospital. Secondary outcomes included 30-day event rates for readmissions, as well as all-cause and PE-related mortality, and serious adverse events.

### Statistical Analysis

For primary and secondary outcomes, a two-sided hypothesis with *P*-value of less than 0.05 was considered to indicate statistical significance. Because subgroup analyses in the present study were exploratory, the *P*-values were not adjusted for multiple comparisons and should be interpreted with caution. Comparisons were made with the use of the *t*-test, the Mann–Whitney U test, Fisher’s exact test, or the chi-squared test, as appropriate. The statistical analyses were performed with the use of the SPSS software package (version 26.0, SPSS) and Stata (version 16.1; StataCorp, College Station, TX, United States).

## Results

A total of 651 patients underwent screening, 500 patients underwent randomization and 498 were included in the modified intention-to-treat analysis – 249 patients were assigned to the intervention group and 249 to the control group. The mean age was 66 years and less than 50% of the patients were women.

Of the 249 patients assigned to the intervention group, 60 (24%) were classified as low-, 159 (64%) as intermediate-low, and 30 (12%) as intermediate-high risk. Among 249 patients who were assigned to the control group, 66 (27%; difference 2.4%; 95% confidence interval [**CI**], −5.5–10.3%) were low-, 170 (68%; difference 4.4%; 95% CI, −4.2–12.9%) intermediate-low, and 13 (5%; difference 6.8%; 95% CI, 1.6–12.2%) intermediate-high risk. Both in the low-risk and in the intermediate-high risk group of patients, the demographic and clinical characteristics of the patients at baseline did not differ between the two trial (intervention and control) groups (Table 1).

**TABLE 1:** Baseline characteristics of the patients.

Characteristic	Low-risk		Intermediate-high risk	
	Intervention group (*N* = 60)	Control group (*N* = 66)	Intervention group (*N* = 30)	Control group (*N* = 13)
**Age – yr**				
Mean	55.1	56.0	73.2	73.7
Range	19–80	21–79	47–92	51–89
**Sex – no. (%)**				
Male	35 (58)	34 (52)	15 (50)	6 (46)
Female	25 (42)	32 (48)	15 (50)	7 (54)
**Medical history – no. (%)**				
Previous VTE	13 (22)	10 (15)	5 (17)	0 (0)
Cancer †	0 (0)	0 (0)	2 (6.7)	3 (23)
Recent surgery ‡	10 (17)	12 (18)	5 (17)	0 (0)
Immobilization §	9 (15)	4 (6.1)	6 (20)	0 (0)
Chronic lung disease	0 (0)	0 (0)	5 (17)	0 (0)
Congestive heart failure	0 (0)	0 (0)	4 (13)	2 (15)
Recent major bleeding	0 (0)	0 (0)	1 (3.3)	0 (0)
**Symptoms – no. (%)**				
Dyspnea	40 (67)	50 (76)	26 (87)	11 (85)
Chest pain	36 (60)	44 (67)	17 (57)	4 (31)
Hemoptysis	8 (13)	7 (11)	1 (3.3)	0 (0)
Syncope	3 (5.0)	7 (11)	8 (27)	5 (38)
**Systolic blood pressure – mm Hg**				
Mean	136.3	139.2	132.7	133.7
95% Confidence interval	131.5–141.2	135.0–143.3	124.4–140.9	120.3–147.0
**Heart rate – beats/min**				
Mean	80.7	82.5	106.2	102.5
95% Confidence interval	77.5–83.9	78.9–86.0	98.6–113.9	90.6–114.3
**Arterial oxyhemoglobin saturation -%**				
Mean	95.5	96.1	90.5	90.1
95% Confidence interval	94.7–96.3	95.3–96.9	87.8–93.3	85.9–94.4
**Hemoglobin – g/dl**				
Mean	13.7	13.8	14.1	13.8
95% Confidence interval	13.2–14.2	13.4–14.3	13.5–14.8	12.8–14.9
**Serum creatinine – mg/dl**				
Mean	0.9	0.9	1.1	1.0
95% Confidence interval	0.8–0.9	0.8–1.0	1.0–1.2	0.9–1.1
**Medications for the acute episode – no. (%)**				
LMWH	60 (100)	61 (92.4)	30 (100)	13 (100)
Unfractionated heparin	0 (0)	2 (3.0)	0 (0)	0 (0)
Fondaparinux	0	1 (1.5)	0 (0)	0 (0)
Direct oral anticoagulants	0 (0)	2 (3.0)	0 (0)	0 (0)

*VTE, venous thromboembolism; LMWH, low-molecular-weight heparin.*

*†Active or under treatment in the last year.*

*‡ In the previous month.*

*§Immobilized patients defined as non-surgical patients who had been immobilized (i.e., total bed rest with bathroom privileges) for ≥ 4 days in the month prior to PE diagnosis.*

In the low-risk group according to the ESC criteria, the mean (±SD) time from randomization to the initiation of mobilization was 0.1 ± 0.3 days in the intervention group vs. 1.0 ± 0.7 days in the control group (mean difference, −1.0 days; 95% CI, −0.8 to −1.2 days; *P* < 0.001). In the intermediate-high risk group, the mean (±SD) time from randomization to the initiation of mobilization was 1.6 ± 0.9 days in the intervention group vs. 1.4 ± 1.0 days in the control group (mean difference, 0.2 days; 95% CI, −0.4 to 0.8 days).

### Outcomes

**FIGURE 1:**
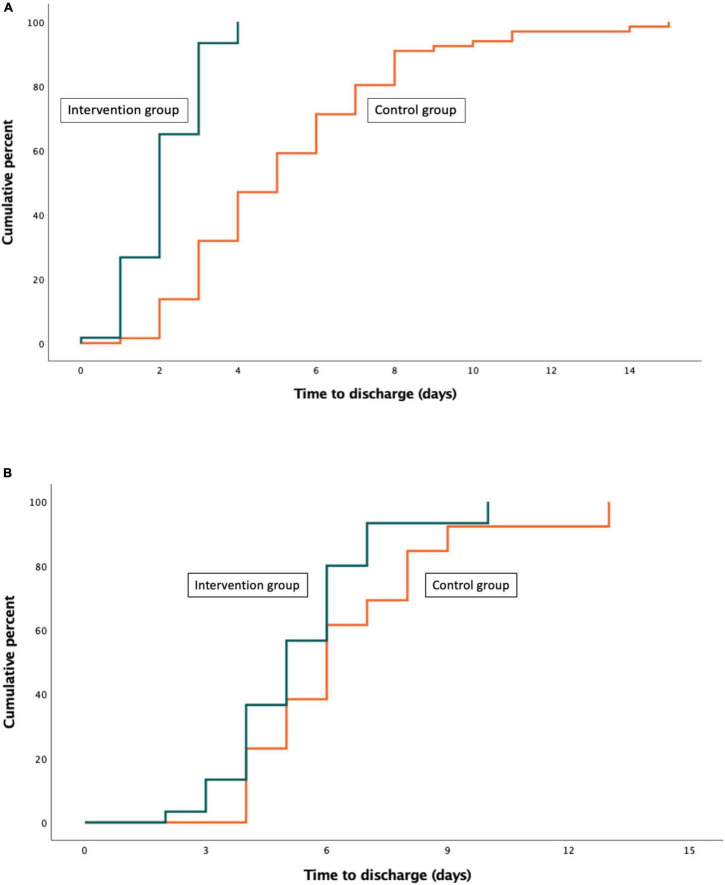
Cumulative frequency distribution curve for the time to discharge of patients in the intervention group as compared with those in the control group. **(A)** Low-risk **(B)** Intermediate-high risk.

Table 2 summarizes the outcomes for study patients according to the subgroup of risk classification. For patients in the low-risk category, the mean LOS was 2.1 (±0.9) days among those randomized to the intervention group vs. 5.3 (±2.9) days among those randomized to the control group (mean difference, −3.2 days; 95% CI, −2.4 to −3.9 days; *P* < 0.001) (Figure 1A). For patients in the intermediate-high risk category, the mean LOS was 5.3 (±1.8) days among those randomized to the intervention group vs. 6.5 (±2.5) days among those randomized to the control group (mean difference, −1.2 days; 95% CI, −2.6 to 0.1 days; *P* = 0.08) (Figure 1B).

**TABLE 2:** Outcomes according to subgroups of risk stratification.

Outcomes	Intervention group	Control group	Difference or relative risk (95% CI)†
**Low-risk patients**			
Length of hospital stay - days Mean 95% confidence interval	2.1 1.9–2.4	5.3 4.6–6.0	−3.2 (−2.4 to −3.9)
30-day readmission rate - no. (%)	0 (0)	2 (3.0)	–
30-day all-cause mortality - no. (%)	0 (0)	0 (0)	–
30-day PE-related mortality - no. (%)	0 (0)	0 (0)	–
30-day serious adverse events - no. (%)	0 (0)	1 (1.5)	–
**Intermediate-high risk patients**			
Length of hospital stay - days Mean 95% confidence interval	5.3 4.6–6.0	6.5 5.0–8.1	−1.2 (−2.6 to 0.1)
30-day readmission rate - no. (%)	1 (3.3)	1 (7.7)	0.41 (0.02 to 7.17)
30-day all-cause mortality - no. (%)	2 (6.7)	1 (7.7)	0.86 (0.07 to 10.38)
30-day PE-related mortality - no. (%)	2 (6.7)	1 (7.7)	0.86 (0.07 to 10.38)
30-day serious adverse events - no. (%)	5 (16.7)	2 (15.4)	1.10 (0.18 to 6.57)

*CI, confidence interval; PE, pulmonary embolism.*

*†Difference (intervention – control) is shown for means. Relative risk (intervention:control) is shown for percentages.*

Thirty-day follow-up data were available for all patients. Among low-risk patients, none of them died during follow-up. Thirty-day all-cause readmission rates were similarly low in the intervention and control groups (0 vs. 3.0%; difference, −3.0%; 95% CI, −11.5 to 4.9%). Thirty-day severe adverse events (0 vs. 1.5%; difference, −1.5%; 95% CI, −9.3 to 6.1%) were not significantly different in the intervention and control groups (Table 2).

Among intermediate-high risk patients, all-cause readmission rates were similar in the intervention and control groups. Thirty-day all-cause (6.7 vs. 7.7%; relative risk, 0.86; 95% CI, 0.07 to 10.38), PE-related mortality (6.7 vs. 7.7%; relative risk, 0.86; 95% CI, 0.07 to 10.38), or severe adverse events (16.7 vs. 15.4%; relative risk, 1.10; 95% CI, 0.18 to 6.57) were not significantly different in the intervention and control groups (Table 2).

## Discussion

This subanalysis of a multicenter randomized trial showed that the use of an standardized prognostic algorithm, compared with usual care, identifies a significantly higher proportion of patients with intermediate-high risk PE. The use of specific criteria for mobilization and discharge was effective and safe in reducing the LOS for subgroups of low- and intermediate-high risk acute PE.

The recent HOME-PE study showed that the HESTIA rule and the sPESI score were similar regarding the proportion of normotensive patients with acute PE treated at home (11). Compared to that trial, IPEP identified fewer patients as low-risk with the sPESI. Possible reasons for this discrepancy are differences in design, hospital settings and patient characteristics. Conversely, the 25% rate of patients with an sPESI of 0 points was similar to the rate in two other recent studies (19, 20). Compared to previous studies (11, 21), our trial used a heart rate less than 100/min as a predefined criterion for discharge. Though the sPESI (which uses a heart rate cutoff of 110/min) has been reported to be helpful to identify low-risk PE patients who might be suitable for home therapy, recent data suggest that a lower heart rate at admission might portend a more favorable prognosis with respect to mortality (22). In fact, none of the low-risk patients in the intervention arm experienced short-term complications. Nevertheless, a prognostic guided management still reduced the LOS by 3 days among low-risk patients, and the mean LOS in the intervention group was not different to that among patients who enrolled the clinical trials of outpatient therapy (11, 21, 22).

An interesting finding of our study is that the use of a standardized prognostic pathway, compared with usual care, identified a significantly higher proportion of patients at intermediate-high risk for short-term complications. This is particularly important since approximately 5% of these patients might deteriorate after diagnosis and initiation of therapy, and require monitoring over the 1st h or days (17). If there was no clinical deterioration within the first 48 h of bed rest, the trial managed intermediate-high risk patients the same way as intermediate-low risk patients (16). With this strategy, the rate of complications was low, and the LOS was reduced by 1.2 days among these intermediate-high risk patients.

Our study has limitations. This was a *post-hoc* analysis, and risk stratification of patients randomly assigned to usual care was done retrospectively. However, all potentially relevant baseline parameters had been documented in the original case report forms of IPEP. Despite the large number of patients enrolled in the trial, the relatively small sample size of the risk subgroups lowered the statistical power of the study. In addition, IPEP was not formally powered to compare the rate of adverse events in the subgroups of risk, but the very low rate of complications reinforces the validity of our approach. We believe that our results could help to generate clinically relevant hypotheses and provide the background for the design of future trials. Finally, the impact of the overall intervention would likely depend on what represents standard of care in a particular institution. This varies widely within and between countries, depending on resourcing of VTE care and awareness of risk stratification strategies.

## Conclusion

In conclusion, this analysis shows that the use of a prognostic assessment and management pathway, compared to usual care, is effective in reducing LOS both in the low- and the intermediate-high risk subgroups. The efficacy and safety of differentiated criteria for mobilization and discharge according to the subgroup of risk stratification are important remaining questions that should be addressed in subsequent studies.

## Data Availability Statement

The raw data supporting the conclusions of this article will be made available by the authors, without undue reservation.

## Ethics Statement

The studies involving human participants were reviewed and approved by the Ramon y Cajal Hospital Ethics Committee. The patients/participants provided their written informed consent to participate in this study.

## Author Contributions

DJ and JL: concept and design. DJ, CR, BP, AP, LJ-P, RL-R, PR-A, AG-O, BB, and JL: acquisition, analysis, or interpretation of data, statistical analysis, and critical revision of the manuscript for important intellectual content. DJ, CR, BB, and JL: drafting of the manuscript. DJ: obtain funding, study supervision, and had full access to all of the data in the study and take responsibility for the integrity of the data and the accuracy of the data analysis. All authors contributed to the article and approved the submitted version.

## Conflict of Interest

DJ has served as an advisor or consultant for Bayer HealthCare Pharmaceuticals, Boehringer Ingelheim, Bristol-Myers Squibb, Daiichi Sankyo, Leo Pharma, Pfizer, ROVI, and Sanofi; served as a speaker or a member of a speakers’ bureau for Bayer HealthCare Pharmaceuticals, Boehringer Ingelheim, Bristol-Myers Squibb, Daiichi Sankyo, Leo Pharma, ROVI, and Sanofi; received grants for clinical research from Daiichi Sankyo, Sanofi, and ROVI. LJ-P has served as an advisor or consultant for Actelion Pharmaceuticals, Bayer HealthCare Pharmaceuticals, Leo Pharma, Menarini, Pfizer, and ROVI. PR-A has served as an advisor or consultant for Leo Pharma and Viatris; served as a speaker or a member of a speakers’ bureau for Bristol-Myers Squibb, Pfizer, Daiichi Sankyo, ROVI, and Viatris; received grants for clinical research from ROVI. BB is a recipient of the IGNITE Award from the Mary Horrigan Connors Center for Women’s Health and Gender Biology at Brigham and Women’s Hospital and reports that he is a consulting expert, on behalf of the plaintiff, for litigation related to two specific brand models of IVC filters. The remaining authors declare that the research was conducted in the absence of any commercial or financial relationships that could be construed as a potential conflict of interest.

## Publisher’s Note

All claims expressed in this article are solely those of the authors and do not necessarily represent those of their affiliated organizations, or those of the publisher, the editors and the reviewers. Any product that may be evaluated in this article, or claim that may be made by its manufacturer, is not guaranteed or endorsed by the publisher.

## Supplementary Material

Supplementary Figure 1 Prognostic assessment and management pathway.
