# Supplementary material for: Effect of Prognostic Guided Management of Patients With Acute Pulmonary Embolism According to the European Society of Cardiology Risk Stratification Model
Source: Front Cardiovasc Med. 2022 Apr 12;9:872115. doi: 10.3389/fcvm.2022.872115 (PMC9039515; doi:10.3389/fcvm.2022.872115)
Supplement: Supplementary Figure 1 — Prognostic assessment and management pathway. [file Image_1.pdf]

Figure S1.

PE, pulmonary embolism

PESI, Pulmonary Embolism Severity Index

RV, right ventricle

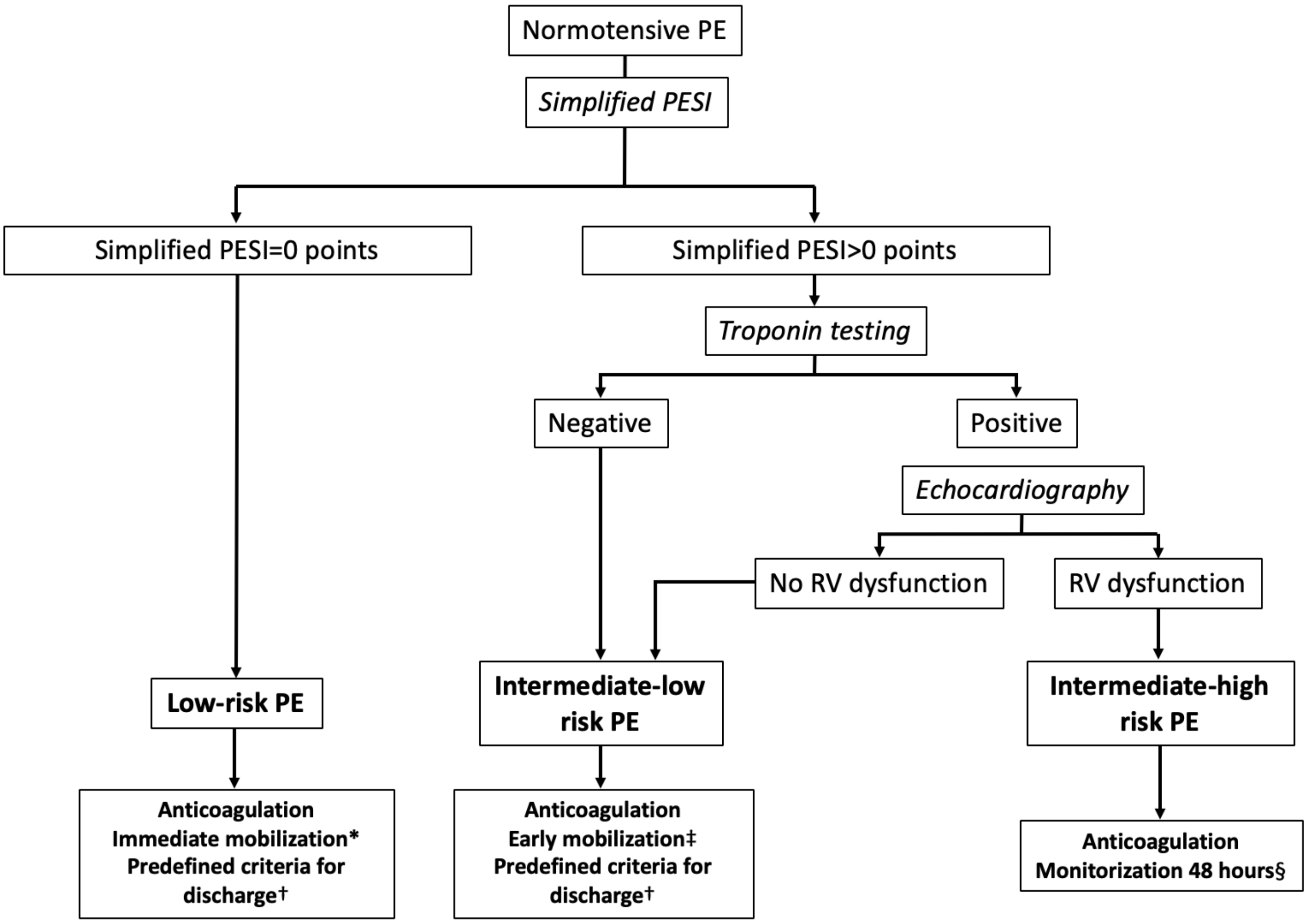

Abbreviations: PE, pulmonary embolism; PESI, Pulmonary Embolism Severity Index; RV, right ventricle.

* Immediate mobilization was defined as ambulation for at least 20 minutes the first morning after randomization.

† Predefined criteria for discharge were meeting criteria for mobilization and adequate vital signs (systolic blood pressure >100 mm Hg, heart rate <100/min, and pulse oximetry >90%).

‡ Early mobilization was defined as ambulation for at least 20 minutes from the second morning after randomization, when they met the following objective criteria: systolic blood pressure >100 mm Hg, heart rate <100/min, and pulse oximetry >90%.

§ If there was no clinical deterioration within the first 48 hours, intermediate-high risk patients were managed the same way as intermediate-low risk patients.
